# The Relationship Between Serum Vitamin D Levels and the First-Attempt Success of the Epley Maneuver in Posterior Canal Benign Paroxysmal Positional Vertigo (PC-BPPV)

**DOI:** 10.3390/jcm15197365

**Published:** 2026-09-22

**Authors:** Akif Sinan Bilgen, Ali Yalçindağ, Özlem Akkoca, Gülşah Çikrikçi IŞIK, Sami Engin Muz, Yüce Islamoğlu, Furkan Yilmaz, Şeyda Gedikaslan Taşdemir

**Affiliations:** 1 Department of Otorhinolaryngology, Medipol Pendik Hospital, Istanbul Medipol University, Istanbul 34893, Turkey; 2 Department of Biochemistry, Ankara Training and Research Hospital, University of Health Sciences, Ankara 06230, Turkey; 3 Department of Otorhinolaryngology, Ankara Training and Research Hospital, University of Health Sciences, Ankara 06230, Turkey; 4 Department of Emergency Medicine, Ankara Atatürk Sanatorium Training and Research Hospital, University of Health Sciences, Ankara 06290, Turkey; gulsah8676@gmail.com; 5 Department of Otorhinolaryngology, Ankara Bilkent City Hospital, University of Health Sciences, Ankara 06800, Turkey; 6 Department of Emergency Medicine, Ankara Bilkent City Hospital, University of Health Sciences, Ankara 06800, Turkey; 7 Department of Emergency Medicine, Yenimahalle Training and Research Hospital, Yıldırım Beyazıt Univesity, Ankara 06370, Turkey

**Keywords:** benign paroxysmal positional vertigo, posterior canal BPPV, vitamin D, 25-hydroxyvitamin D, Epley maneuver, canalith repositioning maneuver, treatment outcome

## Abstract

**Background/Objectives:** Vitamin D deficiency has been associated with the occurrence and recurrence of benign paroxysmal positional vertigo (BPPV), but its influence on the immediate success of canalith repositioning maneuvers remains uncertain. This study investigated the relationship between serum vitamin D levels and the first-attempt success of the Epley maneuver in patients with posterior canal BPPV (PC-BPPV). **Methods:** This prospective observational study included 140 consecutive adults with objectively confirmed unilateral PC-BPPV. Serum 25-hydroxyvitamin D levels were measured at presentation before treatment. All patients underwent a standardized Epley maneuver. First-attempt success was defined as complete resolution of positional vertigo and disappearance of positional nystagmus on the Dix–Hallpike test at the one-week follow-up visit. Serum vitamin D levels were analyzed both as a continuous variable and according to laboratory reference ranges: deficiency (<50 nmol/L), insufficiency (50–75 nmol/L), and sufficiency (75–250 nmol/L). **Results:** The Epley maneuver was successful after the first attempt in 74 of 140 patients (52.9%). In the primary, pre-specified analysis, first-attempt success was more frequent among patients with serum vitamin D ≥50 nmol/L than <50 nmol/L (60.9% vs. 46.1%; OR 1.83, 95% CI 0.93–3.59, *p* = 0.079), a difference that did not reach statistical significance. In unadjusted exploratory analyses, patients with first-attempt success had significantly higher median serum vitamin D levels than those with treatment failure (52.2 [IQR, 30.0–68.4] vs. 36.8 [IQR, 21.3–66.1] nmol/L, *p* = 0.029), and success rates numerically increased across the three-tier classification (46.1%, 62.2%, 59.3%), without a clear dose–response pattern, neither the overall comparison (*p* = 0.208) nor pairwise subgroup comparisons reaching statistical significance. In a multivariable model adjusting for age, sex, and month of enrolment, vitamin D remained independently, though modestly, associated with first-attempt success (adjusted OR 1.14 per 10 nmol/L, 95% CI 1.00–1.29). **Conclusions:** The pre-specified primary comparison of first-attempt Epley maneuver success between vitamin D-deficient and non-deficient patients did not reach statistical significance, although the point estimate favored higher success in the non-deficient group (OR 1.83, 95% CI 0.93–3.59). An exploratory, initially unadjusted analysis suggested a positive, modest association between serum vitamin D as a continuous variable and first-attempt success; this association persisted after multivariable adjustment (adjusted OR 1.14, 95% CI 1.00–1.29) but did not follow a clear dose–response pattern across categorical vitamin D thresholds. These findings should be regarded as hypothesis-generating rather than establishing a definitive prognostic role for vitamin D, and larger, adequately powered multicenter studies are needed to confirm them.

## 1. Introduction

Benign paroxysmal positional vertigo (BPPV) is the most prevalent cause of peripheral vertigo, accounting for more than half of all cases. It is characterized by brief episodes of vertigo triggered by specific changes in head position. The underlying mechanism of BPPV involves the displacement of otoconia from the utricular macula into one of the semicircular canals, where they disturb endolymphatic flow and render the affected canal abnormally sensitive to gravitational forces [1]. Among the various subtypes, posterior canal BPPV (PC-BPPV) is the most frequent, representing more than 90% of all BPPV cases.

The Epley maneuver is the most commonly employed treatment for PC-BPPV due to its non-invasive nature, ease of application, repeatability, and the fact that it requires no specialized equipment [2,3]. It was demonstrated that the Epley maneuver is more effective than pharmacological therapy alone, not only in resolving symptoms but also in reducing the recurrence of PC-BPPV [3]. Epley originally reported a success rate exceeding 90% after a single treatment session; however, subsequent studies have shown that the success rate may vary between 74% and 83% [3,4,5]. The efficacy of repositioning maneuvers can be influenced by patients’ underlying medical conditions, among which serum vitamin D_3_ levels have recently attracted significant research interest [6].

Vitamin D plays a critical role in the proper mineralization of otoconia and in maintaining normal otolith function by regulating calcium homeostasis within the vestibular endolymph [7]. The association between vitamin D status and BPPV was first recognized in the early 2010s, and subsequent studies have demonstrated that vitamin D supplementation in patients with deficiency or insufficiency significantly reduces both the number of relapsing individuals and the frequency of recurrences per patient [8,9,10]. However, evidence regarding whether serum vitamin D levels influence the first-attempt success rate of the Epley maneuver remains limited.

Therefore, the present study aims to evaluate the effect of serum vitamin D levels on the first-attempt success rate of the Epley maneuver in patients with posterior canal BPPV, and to determine whether low vitamin D levels are associated with reduced maneuver success.

## 2. Materials and Methods

### 2.1. Study Design

This single-center, prospective observational study was conducted at a training and research hospital between March 2026 and July 2026 following approval by the local ethics committee. Written informed consent was obtained from all participants, and the study was carried out in accordance with the principles of the Declaration of Helsinki.

### 2.2. Participants

Adult patients presenting to the otorhinolaryngology outpatient clinic with complaints of vertigo were evaluated. The diagnosis of posterior canal benign paroxysmal positional vertigo (PC-BPPV) was established based on a compatible clinical history and the observation of positional nystagmus during the Dix–Hallpike test. The study included only patients with unilateral, objectively confirmed BPPV. Patients who exhibited vertigo or nystagmus in both directions during the test or who had additional pathologies identified on neurological examination were excluded. Other exclusion criteria were the use of vitamin D or calcium supplements within the previous three months, the presence of metabolic bone disease, chronic renal or hepatic disorders, malabsorption syndromes, and pregnancy or lactation.

### 2.3. Study Process

Demographic characteristics, clinical history, and the results of the Dix–Hallpike test were recorded on standardized study forms. Venous blood samples for the measurement of serum vitamin D levels were collected at each patient’s initial presentation.

The Epley maneuver was performed for all patients immediately after the diagnosis of PC-BPPV was established. The maneuver was applied in a standardized manner by experienced otorhinolaryngologists; each treatment session consisted of a single Epley maneuver, with each of the four positions held until the associated nystagmus and vertigo had resolved, plus an additional brief period. Positional nystagmus was assessed by direct (naked-eye) observation, without Frenzel goggles or videonystagmography. First-attempt success of the Epley maneuver was defined as the disappearance of nystagmus on the Dix–Hallpike test and the resolution of vertigo symptoms at the one-week follow-up visit.

The one-week interval was chosen to allow sufficient time for resolution of positional vertigo following successful canalith repositioning while limiting the window during which an interim new BPPV episode could confound outcome ascertainment, consistent with the follow-up interval used in comparable studies [11]. Resolution of vertigo symptoms was ascertained by patient report at the follow-up visit, in conjunction with a negative Dix–Hallpike test; non-positional, nonspecific residual dizziness was not systematically assessed as a distinct outcome in this study, and we cannot exclude that some patients classified as achieving first-attempt success experienced residual non-positional dizziness that was not separately captured. Patients whose symptoms persisted at the one-week follow-up underwent a repeat Epley maneuver at that visit; “first-attempt,” “second-attempt,” etc. therefore refer to separate treatment sessions rather than repeated maneuvers within a single session. The clinician performing the one-week follow-up assessment was not informed of the patient’s serum vitamin D result at the time of assessment.

### 2.4. Laboratory Analysis

Serum vitamin D levels were measured using the chemiluminescence immunoassay (CLIA) method on the DiaSorin Liaison^®^ (DiaSorin S.p.A., Saluggia, Italy) analyzer. Based on the laboratory reference ranges for serum vitamin D concentrations, patients were classified into three categories: vitamin D deficiency (<50 nmol/L), vitamin D insufficiency (50–75 nmol/L), and vitamin D sufficiency (75–250 nmol/L).

### 2.5. Sample Size

The sample size was calculated using a two-proportion comparison power analysis with G*Power for Mac OS X (version 3.1.9.2; Universität Düsseldorf, Germany). Based on previous reports, the expected first-attempt success rate of the Epley maneuver was 74% in patients with sufficient vitamin D levels [3], while it was assumed to be 50% in those with vitamin D deficiency. A two-sided Type I error rate of 5% (α = 0.05) and a Type II error rate of 20% (power = 80%) were adopted. The prevalence of vitamin D deficiency in the target population was estimated at 63% [12].

Under these assumptions, the required total sample size was 126 patients, with approximately 79 in the vitamin D-deficient group and 47 in the vitamin D-sufficient group. Considering a potential 10% attrition rate, at least 140 participants were planned to be enrolled.

The assumed 74% first-attempt success rate in vitamin D-sufficient patients was derived directly from Gaur et al. [3]. At the time of protocol design, no published study had reported first-attempt Epley maneuver success rates stratified specifically by vitamin D status; the assumed 50% success rate in deficient patients therefore reflected a conservative, hypothesis-driven estimate of the anticipated reduction in repositioning success associated with vitamin D deficiency, rather than a value derived from a directly comparable source. Notably, a stratified analysis directly comparable to our assumption was not published until after enrollment in the present study had begun: Rao et al. [13] subsequently reported first-week response rates of 35.4%, 48.6%, and 68.9% across deficient, insufficient, and sufficient vitamin D categories—a 33.5-percentage-point range between the extreme categories, closer to our original 24-point assumption than the 14.9-point difference observed in our primary two-group comparison, and illustrating that estimates of this effect size vary considerably across cohorts. This assumption yielded a 24-percentage-point difference, substantially larger than the 14.9-percentage-point difference actually observed between vitamin D-deficient and non-deficient patients; the implications of this discrepancy for the study’s statistical power are discussed in Section Limitations.

### 2.6. Statistical Analysis

All 140 enrolled patients completed the one-week follow-up assessment and were included in the analysis. Statistical analyses were performed using Jamovi software (version 2.6; The Jamovi Project, Sydney, Australia). Quantitative (continuous or discrete) variables were expressed as mean ± standard deviation (SD) or median with interquartile range (IQR), as appropriate, while qualitative (categorical) variables were presented as frequency and percentage [*n* (%)].

For comparisons of continuous variables between two independent groups, the homogeneity of variances was first evaluated using Levene’s test. Since variances were unequal, Welch’s *t*-test was used instead of Student’s *t*-test. The Mann–Whitney U test was applied to non-normally distributed continuous variables. For the Mann–Whitney U test, effect size was expressed as the rank-biserial correlation (r) and as the probability of superiority (AUC), calculated as AUC = U/(n_1_ × n_2_) and r = 2 × AUC − 1; 95% confidence intervals were derived from the normal approximation to the Mann–Whitney U statistic. Categorical variables were compared between groups using the chi-square test.

All statistical tests were two-tailed, and a *p*-value < 0.05 was considered statistically significant.

The primary, pre-specified analysis was the comparison of first-attempt success rates between patients with serum vitamin D below 50 nmol/L and those at or above this threshold, corresponding directly to the two-proportion comparison for which the study was powered (Section 2.5). The three-tier vitamin D classification (deficient/insufficient/sufficient) and the comparison of serum vitamin D as a continuous variable were pre-specified secondary, exploratory analyses undertaken to characterize the shape of the association in greater detail.

To formally assess whether the association between serum vitamin D and first-attempt success was non-linear, a restricted cubic spline model with four knots (5th, 35th, 65th, and 95th percentiles) was fitted and compared with a linear-only model using a likelihood-ratio test.

A multivariable logistic regression model was fitted with first-attempt success as the dependent variable and serum vitamin D (per 10 nmol/L increase), age, sex, and month of enrolment (coded as a continuous term, March–July, reflecting the period of steepest seasonal rise in 25(OH)D) as covariates. Model goodness-of-fit was assessed with the Hosmer–Lemeshow test.

## 3. Results

A total of 140 patients diagnosed with PC-BPPV were included in the study. The mean age of the study population was 55.9 ± 15.7 years and 98 patients (70%) were female while 42 (30%) were male. Positional nystagmus was localized to the right posterior canal in 73 patients (52.1%) and to the left in 67 patients (47.9%). Serum vitamin D levels ranged from 4.2 to 144.0 nmol/L, with a median of 46.3 nmol/L (IQR, 22.8–67.5). According to the predefined cut-off value of 50 nmol/L, 76 patients (54.3%) were classified as vitamin D deficient and 64 (45.7%) as vitamin D sufficient.

The Epley maneuver achieved first-attempt success—defined as resolution of nystagmus on the Dix–Hallpike test and resolution of vertigo symptoms at the one-week follow-up—in 74 patients (52.9%), whereas the remaining 66 patients (47.1%) required at least one additional maneuver. Among these, 60 patients (42.9%) achieved resolution after a second attempt, 4 (2.9%) after a third attempt, and 2 (1.4%) after a fourth attempt. The demographic and clinical characteristics of the study population are summarized in Table 1.

No statistically significant difference was observed between patients with first-attempt success and those with first-attempt failure with respect to age (57.3 ± 17.3 vs. 54.3 ± 13.6 years; Welch’s t = 1.166, *p* = 0.246), sex distribution (female: 67.6% vs. 72.7%; χ^2^ = 0.442, *p* = 0.506), or side of involvement (χ^2^ = 0.289, *p* = 0.591).

In the primary, pre-specified analysis, first-attempt success was achieved in 39 of 64 patients (60.9%, 95% CI 48.7–71.9) with serum vitamin D ≥50 nmol/L compared with 35 of 76 patients (46.1%, 95% CI 35.3–57.2) with vitamin D <50 nmol/L—a difference of +14.9 percentage points (95% CI −1.7 to 30.1)—(OR 1.83, 95% CI 0.93–3.59; χ^2^ = 3.08, *p* = 0.079) (Table 2). This difference, corresponding directly to the comparison for which the study was powered (Section 2.5), did not reach statistical significance, although the point estimate favored higher success among patients with non-deficient vitamin D levels.

In an unadjusted exploratory analysis, serum vitamin D levels, however, differed significantly between the two outcome groups. Patients who achieved first-attempt success had a significantly higher median vitamin D level than those who did not [52.2 (IQR, 30.0–68.4) vs. 36.8 (IQR, 21.3–66.1) nmol/L; Mann–Whitney U = 2965.5, Z = 2.185, *p* = 0.029; rank-biserial r = 0.21 (95% CI 0.02–0.41); probability of superiority (AUC) = 0.61 (95% CI 0.51–0.70)—that is, a randomly selected patient with first-attempt success had a higher vitamin D level than a randomly selected patient with failure in only 61% of pairs. This corresponds to a small-to-moderate effect, and the wide confidence interval indicates the association should be interpreted cautiously] (Table 3).

In a further exploratory analysis, when patients were categorized using the three-tier vitamin D classification (deficient <50, insufficient 50–75, and sufficient >75 nmol/L; see Methods), first-attempt success rates were 46.1%, 62.2%, and 59.3% in the deficient, insufficient, and sufficient groups, respectively. The overall difference across the three groups was not statistically significant (*p* = 0.208) (Table 4). A formal test for non-linearity, comparing a restricted cubic spline model of vitamin D with a linear model by likelihood-ratio test, found no statistically significant evidence of non-linearity (χ^2^ = 3.61, df = 2, *p* = 0.164) (Table 5). We therefore do not interpret the non-monotonic pattern across the three-tier classification as evidence of a genuine non-linear or threshold relationship; it is more parsimoniously attributed to sampling variability across relatively small subgroups (*n* = 27–76) with substantially overlapping confidence intervals. A quartile-based analysis showed a similar, non-significant pattern (34.3%, 57.1%, 65.7%, and 54.3% in Q1–Q4; χ^2^ across quartiles, *p* = 0.059) (Table 5), presented here as a descriptive, hypothesis-generating observation rather than a confirmed finding. Pairwise comparisons between subgroups likewise showed no statistically significant differences. Taken together, the categorical analyses—non-significant across three tiers and lacking a monotonic gradient—do not establish a clear dose–response relationship between vitamin D status and first-attempt success. The pre-specified primary comparison likewise did not demonstrate a statistically significant association, while exploratory unadjusted and adjusted analyses of vitamin D as a continuous variable suggest a possible, modest positive association that warrants confirmation in adequately powered studies.

In a multivariable logistic regression model adjusting for age, sex, and month of enrolment, higher serum vitamin D remained independently associated with first-attempt success (adjusted OR 1.14 per 10 nmol/L, 95% CI 1.00–1.29, *p* = 0.043) (Table 6). The month of enrolment was also independently associated with first-attempt success (adjusted OR 1.56 per month from March to July, 95% CI 1.18–2.07, *p* = 0.002); success rates rose from 37.5% in March to 72.2% in July, a gradient not fully explained by the parallel seasonal rise in vitamin D levels alone. Age and sex were not independently associated with outcome (*p* = 0.863 and *p* = 0.368, respectively). The model showed adequate calibration (Hosmer–Lemeshow *p* = 0.31).

## 4. Discussion

In this prospective observational study of 140 patients with PC-BPPV, first-attempt success of the Epley maneuver was achieved in 52.9% of patients, a rate somewhat lower than the 74–83% range reported in earlier series [3,4,5] but broadly consistent with more recent cohorts that have specifically examined serum vitamin D status as a prognostic factor [13,14]. In the primary, pre-specified analysis, first-attempt success was more common among patients with non-deficient than deficient vitamin D status (60.9% vs. 46.1%; OR 1.83, 95% CI 0.93–3.59, *p* = 0.079), a difference that did not reach statistical significance, although the point estimate and confidence interval favored higher success with higher vitamin D. In exploratory analyses, serum vitamin D level, analyzed as a continuous variable, was significantly higher among patients who achieved first-attempt success than among those who did not (median 52.2 vs. 36.8 nmol/L; *p* = 0.029; rank-biserial r = 0.21, 95% CI 0.02–0.41), a small-to-moderate and imprecisely estimated effect. Success rates likewise increased numerically across the three-tier vitamin D classification (46.1%, 62.2%, and 59.3% in the deficient, insufficient, and sufficient groups), but neither the overall comparison (*p* = 0.208) nor any pairwise subgroup comparison reached statistical significance; taken together with the absence of a monotonic gradient across categories, these data do not establish a clear dose–response relationship, and a formal test found no evidence that this pattern reflects a genuine non-linear relationship.

These findings are consistent with, but more modest than, those recently reported by Rao et al. who found a significantly graded first-week response rate across three vitamin D categories (35.4% in deficient, 48.6% in insufficient, and 68.9% in sufficient patients; *p* = 0.002) in a larger cohort of 286 patients, with an adjusted odds ratio of 1.49 (95% CI, 1.20–1.85) per 5 ng/mL increase in 25-hydroxyvitamin D [13]. The larger sample size and multivariable adjustment in that study likely provided greater statistical power than the present univariate comparison, which may explain why our categorical analysis fell short of significance despite pointing in the same direction. Similarly, Joo et al. reported that lower serum 25(OH)D was independently associated with refractory BPPV (defined as requiring three or more repositioning attempts), although this association was subtype-dependent and weakened after adjustment for interaction effects [14]. In our cohort, a restricted cubic spline model formally tested for non-linearity in the vitamin D–outcome relationship and found no statistically significant evidence of departure from linearity (*p* = 0.164); the apparent non-monotonic pattern across the three-tier classification is therefore attributable to sampling variability rather than a genuine non-linear effect. We note, however, that the interquartile ranges in Table 3 show near-identical upper bounds between outcome groups (68.4 vs. 66.1 nmol/L) alongside more divergent lower bounds (30.0 vs. 21.3 nmol/L), hinting that the association may be concentrated in the vitamin D-deficient range; this observation is hypothesis-generating and would require formal threshold/changepoint testing in a larger, adequately powered cohort.

In a multivariable model adjusting for age, sex, and month of enrolment, the association between vitamin D and first-attempt success persisted but remained modest and imprecisely estimated (adjusted OR 1.14 per 10 nmol/L, 95% CI 1.00–1.29, a confidence interval that only narrowly excluded the null). Month of enrolment was itself a stronger independent predictor (adjusted OR 1.56 per month, 95% CI 1.18–2.07), with success rising from 37.5% in March to 72.2% in July—a gradient only partly explained by the parallel seasonal rise in vitamin D, raising the possibility that other unmeasured seasonal or practice-related factors contributed to the outcome. Together, these findings suggest that vitamin D may play a modest role in first-attempt Epley success, but the present data do not establish a definitive prognostic role or a clear dose–response relationship.

The biological plausibility of these findings is supported by the established role of vitamin D in regulating calcium homeostasis within the vestibular endolymph and in maintaining the structural integrity of otoconia [7]. Impaired otoconial mineralization secondary to vitamin D deficiency has been proposed as a mechanism that could both predispose to otoconial detachment and delay the resolution of vertigo after repositioning, as free-floating debris may be less readily cleared or may re-aggregate more easily when calcium metabolism is disturbed [15]. This mechanism is further supported by the broader literature linking low vitamin D to BPPV occurrence and recurrence: a recent systematic review and meta-analysis of 60 studies (16,368 participants) found significantly lower vitamin D levels in patients with BPPV compared with controls, and lower levels still in those with recurrent disease, along with a substantially reduced recurrence risk after vitamin D supplementation (RR = 0.45) [16].

Vitamin D has also been implicated in short-term outcomes beyond first-attempt success. Wu et al. found that lower baseline 25(OH)D was independently associated with moderate-to-severe residual dizziness one week after otherwise successful repositioning, particularly among early-onset female patients, suggesting that vitamin D status may influence multiple dimensions of BPPV recovery, not only the mechanical success of the maneuver itself [11]. Taken together, the available evidence suggests that vitamin D may influence not only the recurrence of BPPV but also early treatment response after canalith repositioning. Nevertheless, the magnitude and clinical relevance of this association remain uncertain and warrant further investigation.

The first-attempt success rate observed in this cohort (52.9%) was lower than the 74–83% range reported in several prior series [3,4,5]. This difference likely reflects, at least in part, methodological factors rather than a true difference in treatment efficacy. First, our definition of success required both resolution of positional nystagmus on formal Dix–Hallpike testing and complete resolution of vertigo symptoms at a one-week follow-up visit—a stricter and more delayed criterion than the immediate post-maneuver nystagmus resolution used to define success in some earlier studies. Second, in our protocol, a treatment session consisted of a single Epley maneuver, with repeat maneuvers performed only at a subsequent visit if symptoms persisted at one week, rather than multiple maneuvers repeated within the same session; this may also lower the apparent single-session success rate relative to protocols permitting immediate re-treatment. Third, positional nystagmus was assessed by direct observation without Frenzel goggles or videonystagmography; the absence of fixation suppression could, in principle, have led to under- or over-detection of subtle nystagmus, and we cannot exclude some resulting misclassification of outcome, though the direction of any such bias is not predictable. The clinician performing the one-week follow-up assessment was not informed of the patient’s vitamin D result, which limits the potential for expectation bias in outcome ascertainment.

### Limitations

First, it was conducted at a single center. Although the enrolled sample size (*n* = 140) matched our a priori calculation for the originally assumed 24-percentage-point difference (74% vs. 50%), the difference actually observed in the primary analysis was substantially smaller (14.9 percentage points; 60.9% vs. 46.1%). Post-hoc calculation indicates that the study had approximately 42% power to detect this observed effect at a two-sided α of 0.05, whereas detecting a difference in this magnitude with 80% power would require approximately 175 patients per group (a total of approximately 350 patients)—nearly 2.5 times the current sample. This mismatch between the assumed and observed effect size, rather than the absolute sample size itself, is the principal driver of the non-significant primary result, and underscores the need for larger, adequately powered studies.

Second, we fitted a multivariable logistic regression model adjusting for age, sex, and month of enrolment; serum vitamin D remained independently associated with first-attempt success after this adjustment (Table 6). However, information on osteoporosis, other comorbidities, symptom duration, and previous history of BPPV was not systematically collected in this cohort, and these potentially relevant confounders could not be included in the adjusted model.

Third, serum vitamin D was measured only at baseline, and information regarding seasonal variation, dietary intake, sunlight exposure, and parathyroid hormone or calcium metabolism was not available. Fourth, positional nystagmus was assessed by direct observation without Frenzel goggles or videonystagmography, which may have reduced sensitivity for detecting nystagmus suppressed by visual fixation and could have contributed to outcome misclassification in either direction. Fifth, residual non-positional dizziness—distinct from positional vertigo—was not systematically assessed as a separate outcome in this cohort. Because vitamin D status has itself been associated with residual dizziness after otherwise successful repositioning [11], our first-attempt success outcome may not fully distinguish mechanical repositioning success from broader contributors to persistent dizziness; future studies should assess these outcomes separately. Finally, the study focused exclusively on the first-attempt success of the Epley maneuver and did not evaluate long-term recurrence or the effects of vitamin D supplementation. Future multicenter studies with larger sample sizes and longitudinal follow-up are needed to clarify the independent contribution of vitamin D to both immediate treatment response and long-term outcomes in patients with PC-BPPV.

## 5. Conclusions

In conclusion, the pre-specified primary comparison of first-attempt Epley maneuver success between vitamin D-deficient and non-deficient patients did not reach statistical significance, although the point estimate favored higher success in the non-deficient group. In unadjusted exploratory analyses, serum vitamin D as a continuous variable was positively associated with first-attempt success, a modest and imprecisely estimated effect that persisted after multivariable adjustment for age, sex, and month of enrolment (adjusted OR 1.14 per 10 nmol/L, 95% CI 1.00–1.29). However, success rates did not follow a clear dose–response pattern across categorical vitamin D thresholds, and a formal test found no evidence of a non-linear relationship. These findings should be regarded as hypothesis-generating rather than establishing vitamin D as a definitive prognostic factor. Larger prospective multicenter studies, adequately powered for the effect size observed here and incorporating a broader set of confounders—including symptom duration, previous BPPV history, and osteoporosis/comorbidity status, which could not be assessed in the present cohort—are warranted to confirm these findings and to determine whether correction of vitamin D deficiency can improve short-term treatment outcomes as well as reduce recurrence.

## Figures and Tables

**Table 1:** Demographic and clinical characteristics of the study population (*n* = 140).

Characteristic	Value (*n* = 140)
Age, years, mean ± SD	55.9 ± 15.7
Sex, *n* (%)	
Female	98 (70.0)
Male	42 (30.0)
Side of involvement, *n* (%)	
Right	73 (52.1)
Left	67 (47.9)
Serum vitamin D, nmol/L, median (IQR)	46.3 (22.8–67.5)
Vitamin D status, *n* (%)	
Deficient (<50 nmol/L)	76 (54.3)
Insufficient (50–75 nmol/L)	37 (26.4)
Sufficient (>75 nmol/L)	27 (19.3)
Epley maneuvers required, *n* (%)	
1	74 (52.9)
2	60 (42.9)
3	4 (2.9)
4	2 (1.4)
First-attempt success, *n* (%)	74 (52.9)

SD, standard deviation; IQR, interquartile range.

**Table 2:** Primary pre-specified analysis: first-attempt success according to the 50 nmol/L vitamin D threshold.

Vitamin D Category	N	First-Attempt Success, *n* (%, 95% CI)	OR (95% CI)	*p*-Value
<50 nmol/L (deficient)	76	35 (46.1, 95% CI 35.3–57.2)	Reference	-
≥50 nmol/L (non-deficient)	64	39 (60.9, 95% CI 48.7–71.9)	1.83 (0.93–3.59)	0.079
Difference (≥50 vs. <50)		+14.9 percentage points (95% CI −1.7 to 30.1)		
Total	140	74 (52.9)		

OR, odds ratio; CI, confidence interval. This comparison corresponds directly to the two-proportion comparison for which the study was powered (Section 2.5). 95% CIs for proportions were calculated with the Wilson score method, the difference CI with the Newcombe method, and the OR CI with the logit method.

**Table 3:** Comparison of demographic, clinical, and laboratory characteristics between patients with first-attempt success and first-attempt failure of the Epley maneuver.

Variable	First-Attempt Success (*n* = 74)	First-Attempt Failure (*n* = 66)	Test Statistic	Effect Size (95% CI)	*p*-Value
Age, years, mean ± SD	57.3 ± 17.3	54.3 ± 13.6	t = 1.166		0.246
Sex, *n* (%)			χ^2^ = 0.442		0.506
Female	50 (67.6)	48 (72.7)			
Male	24 (32.4)	18 (27.3)			
Side of involvement, *n* (%)			χ^2^ = 0.289		0.591
Right	37 (50.0)	36 (54.5)			
Left	37 (50.0)	30 (45.5)			
Serum vitamin D, nmol/L, median (IQR)	52.2 (30.0–68.4)	36.8 (21.3–66.1)	U = 2965.5	r = 0.21 (0.02–0.41); AUC = 0.61 (0.51–0.70)	0.029 *

SD, standard deviation; IQR, interquartile range. Age was compared with Welch’s *t*-test (Levene’s test indicated unequal variances, *p* = 0.018); sex and side of involvement were compared with the chi-square test; serum vitamin D level was compared with the Mann–Whitney U test (non-normally distributed, Shapiro–Wilk *p* < 0.001). * *p* < 0.05.

**Table 4:** First-attempt success rate of the Epley maneuver according to a three-tier serum vitamin D classification.

**Vitamin D Category**	***n***	**First-Attempt Success, *n* (%)**	**First-Attempt Failure, *n* (%)**
Deficient (<50 nmol/L)	76	35 (46.1)	41 (53.9)
Insufficient (50–75 nmol/L)	37	23 (62.2)	14 (37.8)
Sufficient (>75 nmol/L)	27	16 (59.3)	11 (40.7)
Total	140	74 (52.9)	66 (47.1)
**Pairwise Comparisons**			
**Comparison**		**χ^2^**	***p*** **-Value**
Deficient (<50 nmol/L) vs. Insufficient (50–75 nmol/L)		2.585	0.108
Insufficient (50–75 nmol/L) vs. Sufficient (>75 nmol/L)		0.055	0.814
Deficient (<50 nmol/L) vs. Sufficient (>75 nmol/L)		1.390	0.238

Overall comparison: chi-square test, *p* = 0.208; comparisons were performed with the chi-square test; none reached statistical significance, including after Bonferroni correction for three comparisons (corrected α = 0.017).

**Table 5:** First-attempt success according to serum vitamin D quartile, and formal test for non-linearity.

Vitamin D Quartile	*n*	Range (nmol/L)	First-Attempt Success, *n* (%, 95% CI)
Q1 (lowest)	35	4.2–22.1	12 (34.3, 95% CI 20.8–50.8)
Q2	35	23.0–45.5	20 (57.1, 95% CI 40.9–72.0)
Q3	35	47.0–67.3	23 (65.7, 95% CI 49.2–79.2)
Q4 (highest)	35	68.0–144.0	19 (54.3, 95% CI 38.2–69.5)

Chi-square across quartiles, *p* = 0.059. A restricted cubic spline model (4 knots) compared with a linear model by likelihood-ratio test found no statistically significant evidence of non-linearity (χ^2^ = 3.61, df = 2, *p* = 0.164).

**Table 6:** Multivariable logistic regression for first-attempt success of the Epley maneuver.

Predictor	Adjusted OR (95% CI)	*p*-Value
Serum vitamin D, per 10 nmol/L)	1.14 (1.00–1.29)	0.043
Age, per year	1.00 (0.98–1.03)	0.863
Sex, male vs. female	1.43 (0.66–3.11)	0.368
Month of enrolment, per month (March→July)	1.56 (1.18–2.07)	0.002

*n* = 140 (74 events, 66 non-events); model likelihood-ratio χ^2^ = 16.62, df = 4, *p* = 0.002; Hosmer–Lemeshow goodness-of-fit *p* = 0.31.

## Data Availability

Data supporting the findings of this study are available from the corresponding author upon reasonable request.

## References

[1] Çıkrıkçı Işık G., Çevik Y., Emektar E., Çorbacıoğlu Ş.K. (2017). Analysis of vitamin D and calcium levels in benign paroxysmal positional vertigo. Eurasian J. Emerg. Med..

[2] Akağlu E., Okuyucu Ş., Okuyucu E., Melek İ.M., Duman T., Dağlı A.S. (2007). The effectiveness of the Epley maneuver for the treatment of BPPV and the role of nystagmus direction as an early indicator of successful treatment. Kulak Burun Bogaz Ihtis. Derg..

[3] Gaur S., Awasthi S.K., Bhadouriya S.K., Saxena R., Pathak V.K., Bisht M. (2015). Efficacy of Epley’s maneuver in treating BPPV patients: A prospective observational study. Int. J. Otolaryngol..

[4] Kulthaveesup A., Bunnag K. (2023). Comparison of outcomes of the Epley and self-Epley maneuvers in PC-BPPV: A randomized controlled trial. Am. J. Otolaryngol..

[5] Fyrmpas G., Barkoulas E., Haidich A.B., Tsalighopoulos M. (2013). Vertigo during the Epley maneuver and success rate in patients with BPPV. Eur. Arch. Otorhinolaryngol..

[6] Çelik E.K., Öner F., Akay H.G. (2024). Do single-session Epley maneuvers treat benign paroxysmal positional vertigo?. Ann. Saudi Med..

[7] Talaat H.S., Kabel A.M., Khaliel L.H., Abuhadied G., El-Naga H.A., Talaat A.S. (2016). Reduction of recurrence rate of benign paroxysmal positional vertigo by treatment of severe vitamin D deficiency. Auris Nasus Larynx.

[8] Rhim G., Kim M.J. (2024). Vitamin D supplementation and recurrence of benign paroxysmal positional vertigo. Nutrients.

[9] Jeong S.H., Kim J.S., Kim H.J., Choi J.Y., Koo J.W., Choi K.D., Park J.Y., Lee S.H., Choi S.Y., Oh S.Y. (2020). Prevention of benign paroxysmal positional vertigo with vitamin D supplementation: A randomized trial. Neurology.

[10] Huang X., Chua K.W., Moh S.P.S., Yuen H.W., Low D.Y.M., Anaikatti P., Iqbal A., Rosario B.H. (2025). Falls and physical function in older patients with benign paroxysmal positional vertigo: Findings from a placebo-controlled, double-blind randomized control trial investigating the efficacy of vitamin D treatment in lowering the recurrence rate of BPPV. Aging Clin. Exp. Res..

[11] Wu J., Jiang C.Y., Bai Y.X., Xu Q., Sun X., Pan H., Shu L., Liu J.R., Chen W. (2023). Effect of the serum 25-hydroxyvitamin D level on risk for short-term residual dizziness after successful repositioning in benign paroxysmal positional vertigo stratified by sex and onset age. Front. Neurol..

[12] Alpdemir M., Alpdemir M.F. (2019). Vitamin D deficiency status in Turkey: A meta-analysis. Int. J. Med. Biochem..

[13] Rao Z., Guo R. (2026). Association between serum 25-hydroxyvitamin D levels and prognosis in benign paroxysmal positional vertigo. Front. Nutr..

[14] Joo H.A., Han S.A., Kim J., Park I.S., Kwak M.Y. (2026). Risk factors for refractory BPPV: Subtype-dependent association of serum 25-hydroxyvitamin D. Front. Neurol..

[15] Abdelmaksoud A.A., Fahim D.F.M., Bazeed S.E.S., Alemam M.F., Aref Z.F. (2021). Relation between vitamin D deficiency and benign paroxysmal positional vertigo. Sci. Rep..

[16] Li Y., Gao P., Ding R., Xu Y., Wang Z., Pei X., Li L. (2025). Association between vitamin D, vitamin D supplementation and benign paroxysmal positional vertigo: A systematic review and meta-analysis. Front. Neurol..

